# Increasing Doxorubicin Loading in Lipid-Shelled Perfluoropropane Nanobubbles *via* a Simple Deprotonation Strategy

**DOI:** 10.3389/fphar.2020.00644

**Published:** 2020-05-12

**Authors:** Pinunta Nittayacharn, Eric Abenojar, Al De Leon, Dana Wegierak, Agata A. Exner

**Affiliations:** ^1^Department of Biomedical Engineering, Case Western Reserve University, Cleveland, OH, United States; ^2^Department of Radiology, Case Western Reserve University, Cleveland, OH, United States; ^3^Department of Physics, Ryerson University, Toronto, ON, Canada

**Keywords:** nanobubbles, doxorubicin, drug-loading, ultrasound contrast agent, ovarian cancer

## Abstract

Drug delivery to solid tumors using echogenic nanobubbles (NBs) and ultrasound (US) has recently gained significant interest. The approach combines attributes of nanomedicine and the enhanced permeation and retention (EPR) effect with the documented benefits of ultrasound to improve tumor drug distribution and treatment outcomes. However, optimized drug loading strategies, the drug-carrying capacity of NBs and their drug delivery efficiency have not been explored in depth and remain unclear. Here, we report for the first time on the development of a novel deprotonated hydrophobic doxorubicin-loaded C_3_F_8_ nanobubble (hDox-NB) for more effective US-mediated drug delivery. In this study, the size distribution and yield of hDox-NBs were measured *via* resonant mass measurement, while their drug-loading capacity was determined using a centrifugal filter technique. *In vitro* acoustic properties including contrast-imaging enhancement, initial echogenic signal, and decay were assessed and compared to doxorubicin hydrochloride loaded-NBs (Dox.HCl-NBs). In addition, *in vitro* therapeutic efficacy of hDox-NBs was evaluated by cytotoxicity assay in human ovarian cancer cells (OVCAR-3). The results showed that the hDox-NBs were small (300.7 ± 4.6 nm), and the drug loading content was significantly enhanced (2 fold higher) compared to Dox.HCl-NBs. Unexpectedly, the *in vitro* acoustic performance was also improved by inclusion of hDox into NBs. hDox-NB showed higher initial US signal and a reduced signal decay rate compared to Dox.HCl-NBs. Furthermore, hDox-NBs combined with higher intensity US exhibited an excellent therapeutic efficacy in human ovarian cancer cells as shown in a reduction in cell viability. These results suggest that hDox-NBs could be considered as a promising theranostic agent to achieve a more effective noninvasive US-mediated drug delivery for cancer treatment.

## Introduction

Ovarian cancer (OC) is the fifth leading cause of cancer deaths among women in the United States. Because the cancer may be asymptomatic, many patients are diagnosed with metastatic disease (Xiao et al., 2009). Because tumor recurrence after surgical resection is common, most women are subsequently treated with systemic or regional chemotherapy. However, response rates are low, and fewer than 30% of these patients will survive beyond 5 years (Torre et al., 2018). To reduce the toxicity and multi-drug resistance of systemic chemotherapies, several nanoscale drug delivery platforms such as PEGylated liposomal doxorubicin (Doxil or Caelyx), micelles, and dendrimers have been developed for OC treatment and have shown more effective tumor accumulation and efficacy compared to free drug owing to the enhanced permeability and retention (EPR) effect (Sudimack and Lee, 2000; Xiao et al., 2009; Torre et al., 2018). Although passive delivery strategies can mediate some of the clinical systemic chemotherapy problems (such as severe cardiotoxicity associated with free doxorubicin), they nonetheless suffer from several limitations such as poor drug distribution and penetration resulting in an insufficient level of drug accumulation at the target tumor. This remains a big challenge in cancer drug delivery. To improve outcomes of systemic drug delivery to solid tumor, various active delivery approaches for triggering the release of drug from vehicles have been developed such as using temperature, ultraviolet (UV)/near-infrared (NIR) light, magnetic resonance (MR), and ultrasound (US) (Chilkoti et al., 2002; Derfus et al., 2007; Husseini and Pitt, 2008; Fomina et al., 2010; Manzoor et al., 2012). Among these, US is considered as an ideal modality for this purpose because it is widely available, relatively inexpensive, safe from hazardous ionizing radiation, and is a non-invasive module for simultaneous real-time imaging and triggering release from vehicle (Böhmer et al., 2009; Deckers and Moonen, 2010; Zhu et al., 2016). A variety of drug-loaded nanoparticles (NPs) such as liposomes or micelles in combination with therapeutic agents have been used in US-mediated drug delivery approach (Husseini et al., 2002; Pruitt and Pitt, 2002; Kim et al., 2013). However, the clinical application of NPs is limited due to their corresponding high resonant frequency which does not allow them to be easily be visible on clinical US. On the contrary, gas-filled microbubbles (MBs) provide great clinical US signal and have been widely used as US contrast agents (UCAs) (Villanueva et al., 2004; Ferrara et al., 2007; Hernot and Klibanov, 2008; Lee et al., 2017; Wang et al., 2017; de Leon et al., 2018). MBs can induce sonoporation of vasculature and cell membranes resulting in an increasing permeability, local drug release, and penetration (Martin and Dayton, 2013). However, their effectiveness *in vivo* is limited as a blood pool agent due to their large size (1–10 µm) which does not allow extravasation beyond the vasculature. Thus, they cannot take advantage of passive delivery *via* the EPR effect for increased tumor specific delivery and efficient intratumoral penetration, which requires particles with diameter in the range of 400-800 nm (Greish, 2010).

As an alternative, US-mediated smaller gas-filled UCAs such as nanobubbles (NBs) have been proposed to improve drug delivery to the tumor. NBs, with a size of 100–600 nm, allow extravasation outside of the vasculature, which can be facilitated *via* EPR effect resulting in higher accumulation in tumor tissue and potentially enhanced theranostic efficiency. Previously, we have developed a highly stable lipid shell-stabilized perfluoropropane (C_3_F_8_) gas NBs and shown that these NBs have minimal signal decay when insonated continuously *in vitro*, has a longer *in vivo* half-life, and has a delayed onset of *in vivo* signal decay (Abenojar et al., 2019; de Leon et al., 2019). We have expanded the application of the previous NB formulation by incorporating chemotherapeutic drug doxorubicin (Dox.HCl), an anthracycline topoisomerase inhibitor, into the NB shell (Nittayacharn et al., 2018). The Dox.HCl-NBs have been shown to improve drug loading efficiency without sacrificing acoustic properties compared to our first generation of US-mediated Dox.HCl-loaded interpenetrating polymer mesh stabilized NBs (Perera et al., 2017; Nittayacharn et al., 2019). However, there are two main limitations to current drug-loaded NBs: (1) the therapeutic efficiency of drug-loaded bubbles has been limited by the loading capacity of the shell which stabilizes the gas core; and (2) high drug loading can destabilize the bubbles, which can result in insufficient therapeutic effect. Accordingly, the objective of this work is to develop a more effective US-mediated drug delivery system that will serve as a theranostic agent for treatment of ovarian cancer. In this study, we aim to improve drug loading directly into the bubble shell by applying a drug-deprotonation strategy which has previously been used to improve micelle loading (Yoo and Park, 2004; Mohan and Rapoport, 2010; Zhang et al., 2016). To our knowledge, this is the first time the concept of deprotonation will be utilized in UCAs. We hypothesize that the loading efficiency of NB can be increased using deprotonated hydrophophobic Dox (hDox). Accordingly, hDox was prepared and loaded into NBs. Drug encapsulation efficiency, size, concentration, and *in vitro* acoustic properties were characterized and compared to commercially available doxorubicin-loaded NBs (Dox.HCl-NBs). In addition, the therapeutic efficacy of NBs was evaluated in cell culture model of human ovarian cancer cells (OVCAR-3).

## Materials and Methods

### Materials

Lipids including DBPC (1,2-dibehenoyl-sn-glycero-3-phosphocholine), DPPA (1,2 dipalmitoyl-sn-Glycero-3-phosphate), and DPPE (1,2-dipalmitoyl-sn-glycero-3-phosphoethanolamine) were obtained from Avanti Polar Lipids (Pelham, AL), and mPEG-DSPE (1,2-distearoyl-sn-glycero-3-phosphoethanolamine-N-[methoxy(polyethylene glycol)-2000] (ammonium salt)) was obtained from Laysan Lipids (Arab, AL). Doxorubicin hydrochloride (Dox.HCl), triethylamine (TEA), tetrahydrofuran (THF), propylene glycol (PG), and the cell proliferation reagent WST-1 were purchased from Sigma Aldrich (Milwaukee, WI). Glycerol was purchased from Acros Organics (Morris, NJ). Roswell Park Memorial Institute (RPMI) 1640 with 10% fetal bovine serum and 1% penicillin-streptomycin, trypsin-EDTA were purchased from Invitrogen (Grand Island, NY). OVCAR-3, human ovarian carcinoma cells were purchased from ATCC (Manassas, VA).

### Preparation and Characterization of Hydrophobic Doxorubicin (hDox)

Commercial doxorubicin hydrochloride (Dox.HCl) was deprotonated to obtain hydrophobic Dox (hDox). Dox.HCl was dissolved in a mixture of chloroform and methanol (3:2, v/v) and incubated overnight with triethylamine (TEA) at 1:3 molar ratio of Dox to TEA which resulted in deprotonation of the sugar amino group (Shuai et al., 2004; Kim et al., 2008; Liu et al., 2012; Wei et al., 2015; Zhang et al., 2016). After solvent evaporation, the deprotonated Dox (hDox) powder was collected and kept in the freezer. The quality of hDox was evaluated by 1HNMR by comparing the main structure with Dox.HCl. The state of hDox was qualitatively determined by thin layer chromatography (TLC). The samples were dissolved in THF and spotted on silica gel TLC plate (TLC silica gel 60 F254, Merk, Darmstadt, Germany) by microcapillary. The plates were developed in the mobile phase consisting of dichloromethane, methanol, formic acid, and deionized water (82:24:2:1, v/v) and were examined under UV light. Testing was performed at least in triplicate.

### Preparation and Purification of Drug-Loaded Nanobubbles

To formulate drug-loaded NBs, hDox was encapsulated in lipid-shell stabilized octafluoropropane (C_3_F_8_) bubbles, as described previously (de Leon et al., 2019). Briefly, lipids including DBPC, DPPA, DPPE, mPEG-DSPE, and 0.2 wt.% hDox were dissolved in propylene glycol (PG). A mixture of glycerol and phosphate buffer saline (PBS) was added then to the lipid solution, and the air inside a sealed 3 ml vial was replaced with C_3_F_8_. Finally, the vial was shaken on a VialMix shaker (Bristol-Myers Squibb Medical Imaging, Inc., N. Billerica, MA) for 45 s to drive bubble self-assembly. NBs were isolated from the mixture by centrifugation at 50 rcf for 5 mins with the vial inverted. Equivalent NBs with regular doxorubicin (Dox.HCl-NBs) were formulated using the same method. The free drug was separated from the drug-loaded NBs by passing the mixture solution of drug-NBs and free drug over a Sephadex column (Sephadex G-25 in PD-10 desalting column, Sigma-Aldrich, UK). NBs were eluted through the column with PBS (pH 7.4) and the first 3 ml fraction containing NBs was collected for further experiments.

### Drug Loading Content and Encapsulation Efficiency

Drug-loaded NB solution (n=3) was transferred into the ultrafiltration unit with a molecular weight cut-off of 50,000 Da (Vivaspin 20, Sartorious) and centrifuged at 4,000 rpm for 50 min to remove free drug (Nittayacharn et al., 2019). The obtained hDox-NBs solution was lyophilized, weighed and dissolved in mixed solution of PBS and THF (1:1, v/v). The fluorescence of hDox was measured by TECAN plate reader (Infinite M200, Tecan Group Ltd., Switzerland) at an excitation of 495 nm and an emission of 595 nm. Equivalent experiments with Dox.HCl-NBs (n=3) were done using the same methods. The encapsulated drug in NBs was calculated by calibration curve obtained with known amounts of drug dissolved in the same solvent solution. Drug content was expressed as the drug loading content (DLC), percentage of encapsulation efficiency (% EE), and the total amount of drug (μg) in bubbles as follows;

(1) $$\begin{array}{l} \text{DLC}=\frac{\text{Amount of drug in particles}(\mathrm{\mu g})}{\text{Weight of lipids }(\mathrm{mg})} \end{array}$$

(2) $$\begin{array}{l} \%\text{EE}=\frac{\mathrm{Amount of drug in particles}(\mu \text{g})}{\mathrm{Initial feeding drug}(\mu \text{g})}\times 100 \end{array}$$

(3) Total drug in bubbles=Encapsulated drug in particles(μg)×Buoyant particle fraction

### Characterization of NB Morphology, Size, and Concentration

The size distribution, concentration and buoyant mass of NBs were measured using resonant mass measurement (RMM) (Archimedes, Malvern Pananalytical Inc., Westborough, MA, USA) using a calibrated nanosensor (100 nm–2 µm) (Hernandez et al., 2019). Sensors were pre-calibrated using NIST traceable 565 nm polystyrene bead standards (ThermoFisher 4010S, Waltham MA, USA). hDox-NBs were diluted 1:1,000 with phosphate buffered saline (PBS, pH 7.4). A total of 1,000 particles were measured for each trial (n=3). Equivalent experiments with Dox.HCl-NBs were done using the same methods. Bubble morphology was imaged with a transmission electron microscope (TEM; Tecnai™ G^2^ Spirit BioTWIN, FEI Company) operated at 120 kV based on a previously reported method (Owen and Stride, 2015). 10 μl of a dilute suspension of the samples was placed in an inverted position for 1 min on a 400 mesh Formvar^®^-coated copper grid. The sample was then stained by placing it on top of a 20 µl droplet of 2% uranyl acetate for 30 s and the excess was removed. The TEM grid containing the bubble sample was allowed to dry for another 30 min. All the characterizations were carried out in triplicate.

### Stability Under Ultrasound

NBs were diluted in PBS at 1:100 and poured into a tissue mimicking agarose phantom (Abenojar et al., 2019; de Leon et al., 2019) placed directly over an ultrasound transducer (PLT-1204BT). Nonlinear contrast images were continuously acquired using a clinical US scanner (AplioXG SSA-790A, Toshiba Medical Imaging Systems, Otawara-Shi, Japan) *via* contrast harmonic imaging (CHI, 12 MHz, mechanical index 0.1, focus depth of 1.5 cm, 2D gain of 70 dB, dynamic range of 65 dB) at 1 frame per second for 8 min. Raw echo power data was recorded and analyzed using built-in software. Initial signal enhancement, signal decay over time, and percent remaining signal at 8 min were determined from the data. The experiment was carried out in triplicate.

### *In Vitro* Cell Viability

Cytoxicity of free drug against OVCAR-3 cells was evaluated by comparing the half maximal inhibitory concentration (IC_50_) of free Dox.HCl and hDox prior to evaluating the *in vitro* efficiency of drug-loaded NBs. Cells were seeded in 96 well plates at 5x10^4^ cells/ml (200 µl/well) and incubated overnight. Then, cells were incubated with four concentrations of free drugs dissolved in a serum-free RPMI media with 1% DMSO (0.025, 0.25, 1.25, 2.5 µg/ml) for 3 h. After the 3-h incubation, the cells were washed with 100 µl PBS 3 times, and replaced with 200 µl of RPMI media with 10% FBS. Following an additional 72-h incubation, cell viability was determined using a proliferation reagent, WST-1, which is a colorimetric assay for the quantification of cell viability and proliferation based on mitochondrial dehydrogenases caused by the cleavage of the tetrazolium salt WST-1. Cells were incubated with WST-1 (1:10, v/v) for 1 h and the absorbance at 450 nm was measured using TECAN plate reader. To assess the *in vitro* efficiency of drug-loaded NBs, cells were prepared as above and were treated with the following treatment conditions: (1) hDox-NBs +US; (2) hDox-NBs; (3) plain NBs+US; (4) plain NBs, (5) free hDox+US; (6) free hDox; (7) free hDox+plain NBs+US; (8) free hDox+plain NBs. Cells were treated with serum-free RPMI medium only (with US and without US) as a control. The “plain NBs” were not loaded with drug. “Free hDox” refers to unencapsulated drug. hDox-NBs and plain NBs were purified with size exclusion gel chromatography as described in the previous section and further diluted with RPMI media at 1:10 dilution. The concentration of hDox and bubbles in each treatment group was control to be equal at 2 µg/ml of hDox and 8.75x10^8^ particles/ml of bubbles. Each well was filled with 400 µl of treatment solution and wrapped with sterile transparent film dressing (Tegaderm™). For the group with ultrasound (+US), cells were exposed to an unfocused US transducer with an effective radiating area of 2 cm^2^ at 1 MHz, 1.7 W/cm^2^, 100% duty cycle, for 1 min. After treatment and 3-h incubation, cells were washed with 100 µl PBS 3 times, replaced with complete RPMI media. Following a 72-h incubation, cell viability was determined using WST-1 as described above. All experiments regarding the cytotoxic activity were carried out in triplicate.

### Statistical Analysis

In this study, all experiments were repeated in triplicate, and the results were presented as mean ± standard deviation (SD), unless otherwise noted. The results fit a normal distribution. Thus, a one-way analysis of variance (ANOVA) with the Tukey test were used to assess statistical significance between groups. All statistical analyses were performed using Origin. A p value of <0.05 was considered significant, unless otherwise noted.

## Results

### NB Characterization

By using an excess amount of TEA, the protonated Dox.HCl was successfully deprotonated under the basic condition resulting in the formation of the hydrophobic Dox (hDox). To elucidate the state of Dox whether or not it is hydrophobic, polarity of the hDox was analyzed compared to Dox.HCl using TLC plate as shown in **Figure S1A**. The Dox.HCl or hydrophilic Dox presented one spot on the TLC plate with R_f_ of 0.63 while two spots with the larger R_f_ of 0.95 was found for the hDox. The ability of hDox to travel further on the TLC plate confirmed that it has less polarity on the other word it is more hydrophobicity. The 1HNMR spectrum also showed the main structure of hDox is similar to that of Dox.HCl which implied that the deprotonation doesn’t change biological activity and the active part of Dox (**Figure S1B**).

**Figure 1:**
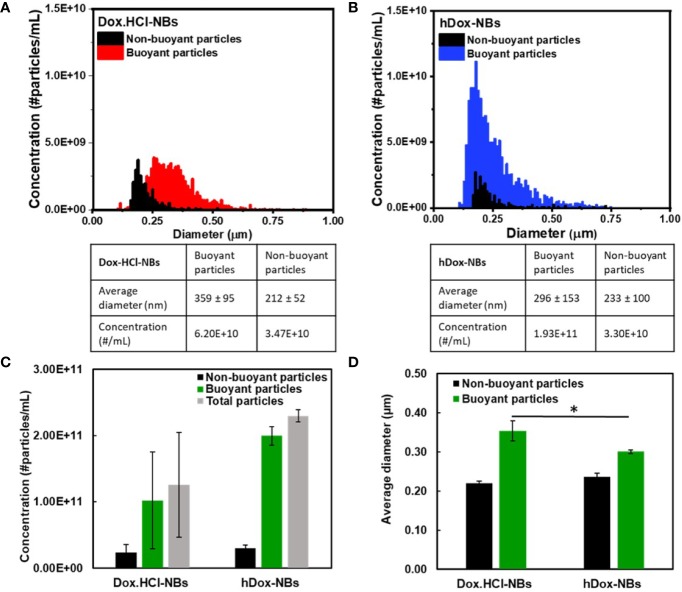
Representative histograms showing the size distribution and concentration of **(A)** Dox.HCl-NBs (n=3) and **(B)** hDox-NBs (n=3), as measured by resonant mass measurement (RMM); The data is summarized to facilitate comparison between groups for both the **(C)** concentration and **(D)** size of buoyant and non-buoyant particles for the two different bubble formulations. Both bubble types were found to have similar concentrations of both buoyant (bubbles) and non-buoyant (solid lipids/aggregates), although the results from Dox.HCl-NBs were more variable and the concentration was on average 50% lower. The results were not statistically significant. The average bubble size of hDox was 25% smaller than Dox.HCl. Asterisk indicates significant difference at p < 0.05.

The average diameter and concentration of the NBs before and after loading with Dox.HCl or hDox were determined by RMM. Results show consistent size of buoyant and non-buoyant particles in the range of 100–600 nm for both formulations, which is on par with the reported size range of nanobubbles as shown in **Figures 1A, B**. Plain NBs showed an average diameter of 280 ± 112 nm while 359 ± 95 and 296 ± 153 nm were observed in Dox.HCl-NBs and hDox-NBs, respectively. hDox loading significantly altered the size of NBs, resulting in 25% smaller size compared to Dox.HCl-NBs (**Figure 1D**). A 10% diameter increase was seen in both formulations after drug loading. Both formulations had a concentration on the order of 10^11^ particles/ml, but the buoyant fraction of hDox-NBs was 50% higher than the Dox.HCl-NBs. The difference was not significantly significant due to the high variability of yield of the Dox.HCl-NBs. The non-buoyant particles, which are likely a combination of micelles and lipid aggregates, were present in both formulations, but with a difference of bubble to micelle/lipid aggregate ratios. However, no significant differences were observed between formulations (**Figure 1C**).

**Figure 2:**
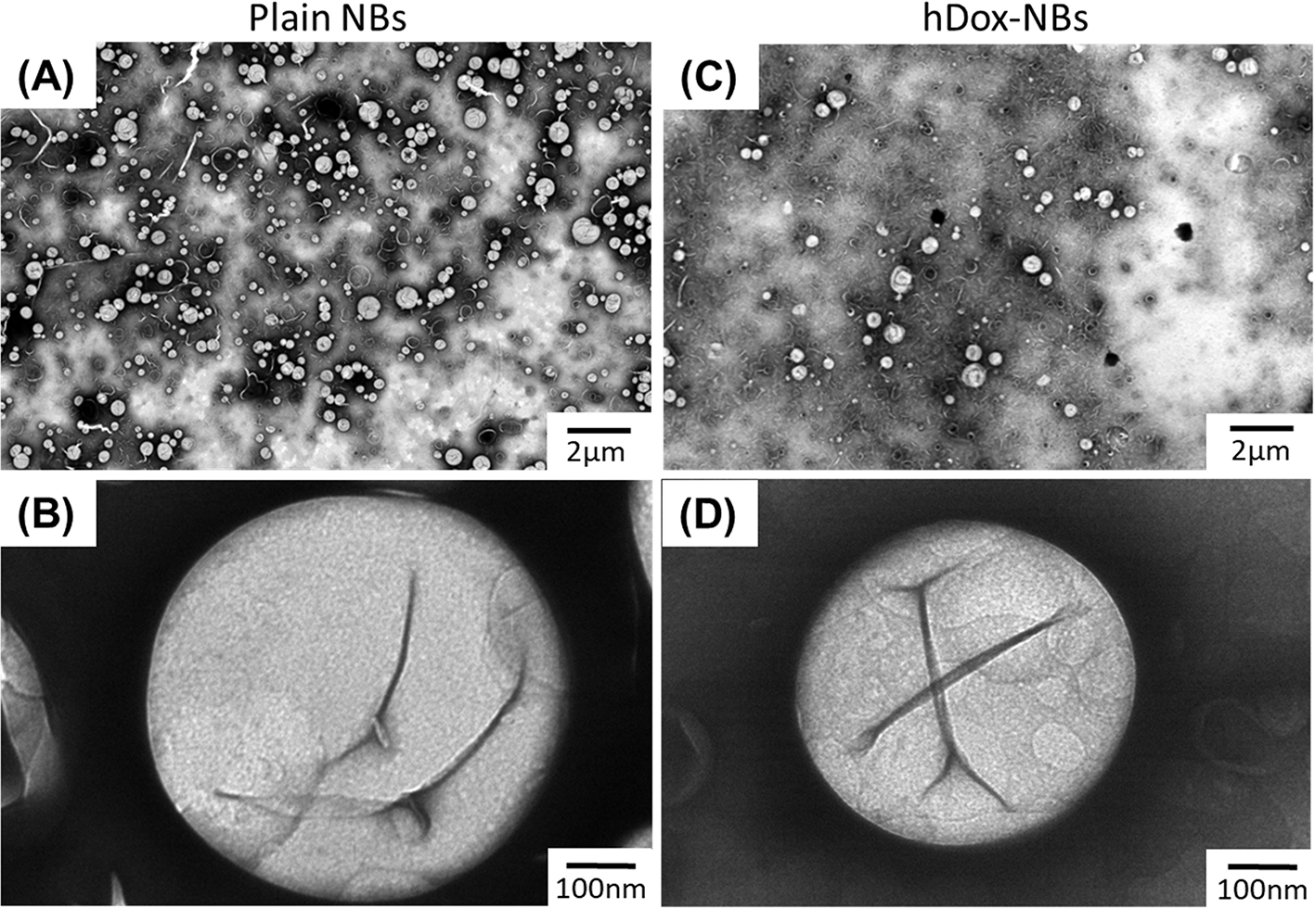
Representative transmission electron microscope (TEM) images showing size and morphology of plain nanobubbles (NBs) or unloaded-NBs **(A**, **B)** and hDox-NBs **(C**, **D)** carried out using uranyl acetate staining. TEM studies were repeated in triplicate. The hDox-NBs show distinct patchy domains and increased ridges and buckles compared to bubbles without Dox.

The morphology of hDox-NBs was also evaluated by transmission electron microscopy (TEM) and compared to that of plain-NBs (**Figure 2**). The TEM image of hDox-NBs clearly showed spherical shape between 200 and 400 nm in diameter, consistent with the NB size distribution obtained from RMM. By Increasing the magnification, the discontinuities caused by folds in the shell was found at the surface of the NBs, which was similar to the phospholipid MB morphology reported in a previous study (Owen and Stride, 2015).

### Dox Loading Content and Encapsulation Efficiency

**Figure 3:**
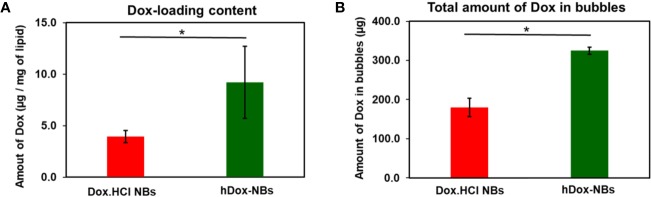
Summary of drug loading into bubbles for hDox (n=3) and Dox.HCl (n=3). **(A)** hDox loading content per milligram of lipid; **(B)** Total hDox amount in bubbles (μg) calculated based on the buoyant particle concentration of both bubble types measured by resonant mass measurement (RMM). Dox in non-buoyant particle fraction was removed from this analysis. Significantly increased drug-loading of hDox can be seen in both test. Error bars represent standard deviation. Asterisk indicates significant difference at significant difference at p < 0.05.

hDox at initial feeding concentration of 2 mg/ml was considered as the optimal concentration as it presented the highest loading content and bubble yield compared to the others (**Figure S3**). The amount of hDox in particles (both bubbles and non-buoyant particles) of Dox.HCl-NBs and hDox-NBs was determined by centrifuge filtration and present as drug loading content (DLC), % EE, and amount of hDox in bubbles as previously described. High DLC and EE was obtained by hDox-NBs. The encapsulation efficiency of hDox-NBs and Dox.HCl-NBs was 18.7 ± 2.0% and 11.4 ± 4.5%, respectively. DLC in hDox-NBs (9.2 ± 3.5 µg) was two times higher than Dox.HCl-NBs (3.9 ± 0.6 µg) as shown in **Figure 3A**. hDox-NBs also had a significantly higher total amount of hDox in bubbles with 324.6 ± 9.2 μg compared to 179.7 ± 23.8 μg in Dox.HCl-NBs (**Figure 3B**).

### Echogenic Performance of hDox-NBs

**Figure 4:**
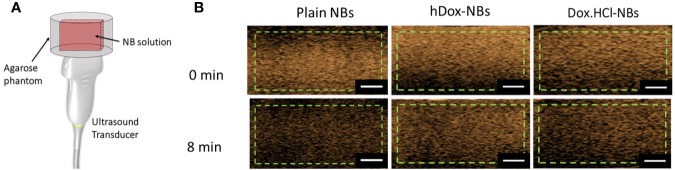
**(A)** Schematic of the ultrasound transducer and agarose phantom with a thin channel (L × W × H = 22 × 1 × 10 mm^3^) where is a sample location; As mentioned previously, the phantom design allows the entire bubble sample to be in the acoustic field, as the width of the slot is the same as the element array. This gives a more accurate measure of nanobubble (NB) stability in the acoustic field. **(B)** Representative ultrasound contrast images for each formulation with the analyzed region of interest (green dashed line). The scale bars are 0.5 cm.

**Figure 5:**
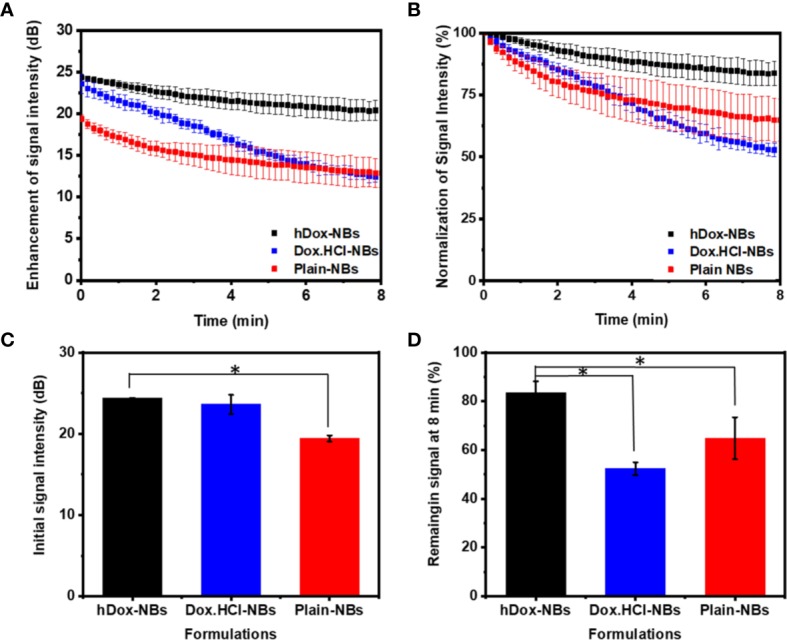
Acoustic properties of hDox-NBs compared to unloaded nanobubbles (NBs) and NBs formulated with Dox.HCl (n=3 for each group). **(A)** Ultrasound signal decay of the three formulations over an 8-min exposure period. While both hDox and Dox.HCl increase the initial backscatter at t=0 compared to plain NBs, the signal decay is more rapid for Dox.HCl-NBs **(B)** Relative ultrasound signal decay rate illustrates the faster decay of Dox.HCl-NBs; Differences in initial ultrasound signal intensity **(C)** and remaining ultrasound signal at 8 min (% of signal at t=0) **(D)**. Error bars represent standard deviation. Asterisk indicates significant difference at p < 0.05.

The *in vitro* stability of hDox-NBs with continuous insonation was evaluated by using tissue-mimicking phantom made from agarose as shown in **Figure 4A** (Hernandez et al., 2019). The representative ultrasound contrast images of Dox-HCl and hDox-NBs showed enhanced nonlinear activity compared to plain NBs (**Figure 4B**). Interestingly, the signal decay rate of hDox-NBs was slower than plain NBs or Dox.HCl-NBs, as shown in **Figures 5A, B**. Although both Dox.HCl and hDox-NB formulations showed a higher initial signal intensity compared to plain NBs (**Figure 5C**), hDox-NBs showed less than 20% of signal loss after 8 min (**Figure 5D**).

### Enhancement of an *In Vitro* Therapeutic Efficacy

The cytotoxicity of free Dox.HCl and hDox at various concentrations was evaluated in order to see the effect of deprotonation on the biological activity. We found that the IC_50_ corresponding to the concentration of the compound that shows 50% of cell viability of both Dox.HCl and hDox is 0.54 and 0.86 µg/ml, respectively (**Figure S6**). *In vitro* therapeutic efficacy of the hDox-NB construct was assessed using a human ovarian carcinoma cell line (OVCAR-3). The US delivery condition of 1 MHz and 1.7 W/cm^2^ at 100% duty cycle with an exposure time of 1 min was used for cell experiments in this work. The cytotoxicity of plain NBs in combination with US at various bubble concentration was first determined as shown in **Figure S7**. At high NB concentration in the range of 86 to 430x10^8^ particles/ml, there was no cytotoxicity. The treatment groups without NB (only cells) showed similar levels of cytotoxicity regardless of the US exposure. The high concentration of bubbles might attenuate the acoustic wave resulting in fewer cavitation events. When the concentration was reduced to 8.75 x10^8^ particles/ml, which is equivalent to the concentration of hDox-NBs used in the following cell viability experiments, the cell viability was at 80%. However, cell viability was drastically reduced and the toxicity was noted again with bubble concentration of 35x10^8^ particles/ml.

**Figure 6:**
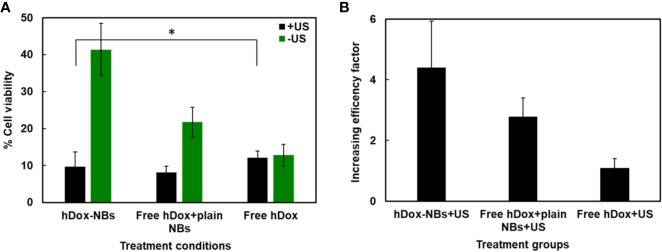
Enhancement of Dox cytotoxicity in OVCAR-3 cells after treatment with hDox-NBs and ultrasound (+US). **(A)** Cell viability of OVCAR-3 cells for different treatments normalized to the untreated control; hDox-NBs have significantly lower toxicity compared to free hDox or free hDox with plain nanobubbles (NBs) when ultrasound is not present. With ultrasound application, the hDox-NBs lead to greater reduction in cell viability compared to free Dox and equivalent reduction compared to Dox co-injected with plain NBs. **(B)** An increasing efficacy factor for each treatment group compared to the group without ultrasound (-US). All experiments were carried out in triplicate. Error bars represent standard deviation. Asterisk indicates significant difference at p < 0.05.

The therapeutic effect of hDox-NBs on OVCAR-3 cells is shown in **Figure 6A**. Without the application of US, free hDox alone shows the highest toxicity. In contrast, hDox-NBs without US showed a nearly 4-fold lower baseline toxicity, which implies that encapsulation of hDox in NBs could be safer to administer *in vivo* with compared to free hDox. When the US was applied, cell viability was decreased over 4-fold with hDox-NBs. In contrast, exposure to cells given free hDox (**Figure 6B**) does not offer additional benefit. Cell viability was also decreased when the US was applied to the plain NB together with free hDox. Free hDox and plain NB also showed significant decrease in cell viability compared to free hDox alone. This is likely the result of sonoporation which causes transient disruption of the cell membrane and increases free drug uptake (Fan et al., 2014; Abdalkader et al., 2017; Helfield et al., 2017; Wang et al., 2018).

## Discussion

We successfully loaded hDox into the shell of our bubbles where the co-localized hDox was observed by the microscopic image (**Figure S2**). Since most of NBs should be below the light diffraction limit, we used larger particles prior to separation of NB population in order to accurately visualize hDox loading on the bubble. The bubble morphology and dox localization in the bubble shell was clearly visualized and was similar to what was previously observed. (Chen et al., 2017; Nittayacharn et al., 2019). To translate hDox-NBs to a clinical application in the future, it may be important to calculate the NB dose compared to typically used MB doses. We estimated the amount of hDox in one bubble to be 162.3 x10^-11^ μg. Doxil, which is the liposomal formulation of doxorubicin hydrochloride (Dox.HCl) at a concentration of 2 mg/ml (per vial), is typically given intravenously at a dose of 50 mg/m^2^ every 4 weeks until disease progression or unacceptable toxicity in ovarian cancer patients whose disease has progressed or recurred after platinum-based chemotherapy. This indicated dose corresponds to a dose of 100 mg for an adult of about 80 kg body weight. The standard clinical dose of DEFINITY^®^ MBs (plain bubbles) is 10 μl/kg that makes 10^10^ MBs for a person of 80 kg (Shelton et al., 2017). To reach a 100 mg dose, about 6 x 10^13^ of hDox-NBs should be administered to the patient. However, because of the 10^3^ decrease in bubble volume when bubble diameter is reduced by an order of magnitude, this NB dose can be achieved with the equivalent total material as needed for the MB dose. Moreover, research shows that DEFINITY^®^ at 1,000 times higher than recommended dose does not produce adverse effects in non-human primates (Lentacker et al., 2010). It is thus likely that a therapeutic dose of drug loaded NBs is clinically feasible and experientially achievable. Furthermore, when considering high NB margination in flow due to lower particle density of NBs (Toy et al., 2011; Cooley et al., 2018), an increased tumor uptake and extravasation *via* concentration gradient will most likely allow us to reduce the clinical dose below 100 mg.

*In vitro* acoustic performance including contrast-imaging enhancement, initial echogenic signal, and decay was greatly improved by inclusion of hDox into NBs. These results suggest that incorporating hDox in the lipid shell stabilizes the NBs and significantly slows gas dissipation from NBs oscillating in the acoustic field. We hypothesize that hDox may represent a similar behavior as cholesterol by altering the flexibility of NB shell, resulting in more compressibility under insonation, less lipid shedding, and lower gas diffusion. The chemical structure of doxorubicin consists of active sites including sugar amino acid group, hydroxyl groups, and ketone groups and largely hydrophobic anthracycline backbone(Blum and Carter, 1974). This structure resembles with cholesterol, which can fill in the gaps between lipid membranes resulting in either an increase or decrease in membrane fluidity by disordering of gel-liquid crystalline phase (Hofsäß et al., 2003; Doxastakis et al., 2005). It is thus possible that, due to the high degree of hydrophobicity, hDox may incorporate more within the hydrocarbon chains of the lipid shell, thus preventing membrane lipids from packing close together. It is also possible that the microviscosity in the phospholipid head group is decreased by hDox (Alves et al., 2017). Accordingly, two interactions involved in hDox loading on NB shell including the hydrophobic interactions with the hydrocarbon chain of the phospholipid and the electrostatic interactions with the negative phosphate group. It is also possible that the degree of hydrophobicity of dox and it stability could influence on the interaction between the dox and lipid shell membrane. Deprotonation of Dox.HCl using TEA does not change this main structure but only removes the HCl at the sugar amino acid group. We found that hDox-NBs where Dox.HCl was deprotonated by TEA was more stable under insonation showing a slower decay rate than hDox-NBs where hydrophobic dox was deprotonated by sodium hydroxide (**Figure S4**).

We also found that the higher signal and slower signal decay were dependent on the amount of encapsulated hDox. An increasing enhancement of US signal was found only at 2 mg/ml of initial feeding hDox concentration (**Figure S5A**). This led us to also investigate the effect of shell composition on bubble surface tension using previously reported methods (Hernandez et al., 2018). One of the most common applications of the lipid solution is stabilization of the gas−water interface by lowering of interfacial tension. Pendant drop tensiometry was used to measure samples of the 0 mg, 0.1, 0.2, and 0.3 mg hDox solutions. In this technique, gravitational pull causes deformations in the shape of suspended droplets and the surface tension is determined from shape fitting of the droplet outline to the Young-Laplace model (Berry et al., 2015). In order to maximize the gravitational deformation of the droplets, samples were discharged from the needle to hold the largest possible, stable droplet (able to remain on the needle tip for a minimum of 15 s). Water was used to show accurate calibration of the system and measurements were collected at 22°C. 10 droplets of each hDox concentration were measured. The membrane surface tension was significantly reduced at only 2 mg/ml hDox loading (**Figure S5B**). This implies that hDox could act as a buffer, increasing the NB membrane fluidity and decreasing fluidity at a certain loading capacity. Together, this evidence is supportive of our hypothesis that the incorporation of hDox on the NB shell membrane may alter the compressibility of NB by adding more robustness to withstand applied US pressure. An in depth mechanism will be investigated in future work.

*In vitro* cytotoxicity of free hDox and hDox-NBs was assessed using a human ovarian carcinoma cell line (OVCAR-3). The biological activity of hDox was shown to be similar to that of Dox.HCl. The drug deprotonation strategy has previously been used to improve micelle loading and has been validated in other hormone-dependent cancers and other cancers including human squamous cell carcinoma (Yoo and Park, 2004); human ovarian cancer (A2780)(Mohan and Rapoport, 2010); and human liver cancer (HepG2)(Zhang et al., 2016). Our results show that the combination therapy of hDox-NBs in the presence of US lead to increased cytotoxicity in comparison to control groups including plain NBs, plain NBs+US, free hDox, free hDox+US, free hDox+plain NBs. Furthermore, the baseline cytotoxicity of hDox-NBs without sonication were lower than toxicity of free Dox with or without ultrasound. The cytotoxicity of plain NBs with and without US was also examined to assess baseline toxicity without drug. We found that at the selected NB concentration and sonication parameters, little to no toxicity was seen. Accordingly, it is likely that hDox-NBs in combination with US are responsible for the observed cytotoxic effects *in vitro*. It is also worth mentioning that perfluoropropane (C_3_F_8_) gas used in this formulation is also not likely to show toxic effects. The gas is used in several clinical applications including in commercially available microbubbles and in clinical vitreoretinal surgery (Kurt et al., 2009; Modi et al., 2017) with only rare adverse effects reported.

## Conclusion

Overall, this work provides evidence that drug loading capacity, acoustic performance, and therapeutic efficacy can be enhanced by simple deprotonation of doxorubicin prior to its loading into lipid-stabilized NBs. These characteristics suggest that hDox-NBs may be a potential tool for more effective and tumor specific drug delivery when combined with molecular targeting in future work. Results from this study will lead to the development of US-mediated drug delivery system as a theranostic agent with the capability of diagnostic and treating metastatic cancer.

## Data Availability Statement

The raw data supporting the conclusions of this article will be made available by the authors, without undue reservation, to any qualified researcher.

## Author Contributions

PN conceptualized the idea, designed the study, completed the main experiments, analyzed the data and wrote the first draft of the paper. EA contributed to study design, nanobubble characterization and TEM imaging. AD participated in conceptualization of the idea and manuscript preparation. DW contributed to study design and carried out the surface tension experiments. AE conceptualized the idea, oversaw study design and implementation, data analysis and manuscript preparation. All authors have reviewed the final version of the manuscript and approved it for publication.

## Funding

This work was supported by the National Institutes of Health (R01EB025741 and R01EB028144) and PN was supported by the Royal Thai Government, Thailand. Support was also provided through MITACS Globalink program.

## Conflict of Interest

The authors declare that the research was conducted in the absence of any commercial or financial relationships that could be construed as a potential conflict of interest.
