# Supplementary material for: Increasing Doxorubicin Loading in Lipid-Shelled Perfluoropropane Nanobubbles via a Simple Deprotonation Strategy
Source: Front Pharmacol. 2020 May 12;11:644. doi: 10.3389/fphar.2020.00644 (PMC7235281; doi:10.3389/fphar.2020.00644)
Supplement: Supplementary file 1 [file DataSheet_1.pdf]

Supplementary Material

Increasing Doxorubicin Loading in Lipid-Shelled Perfluoropropane Nanobubbles via a Simple Deprotonation Strategy

Pinunta Nittayacharn [1], Eric Abenojar [2], Al De Leon [2], Dana Wegierak[3], Agata A. Exner [1,2*]

[1] Department of Biomedical Engineering, Case Western Reserve University, Cleveland, OH, USA

[2] Department of Radiology, Case Western Reserve University, Cleveland, OH, USA.

[3] Department of Physics, Ryerson University, Toronto, Canada.

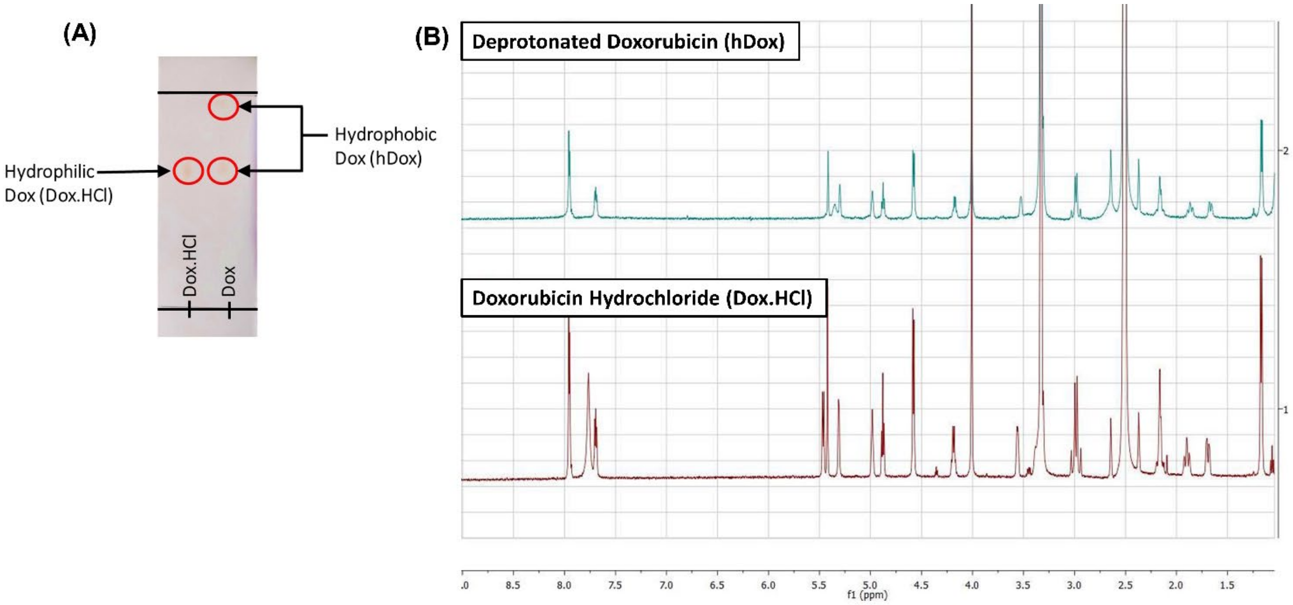

Supplementary Figure 1. (a) TLC plate showing the state of hydrophilic Dox (Dox.HCl, left lane) and hydrophobic Dox (hDox, right lane); (b) NMR spectrum showing the main structure of hDox and Dox.HCl. The analysis shows that the primary structure of Dox remains intact following the deprotonation process.

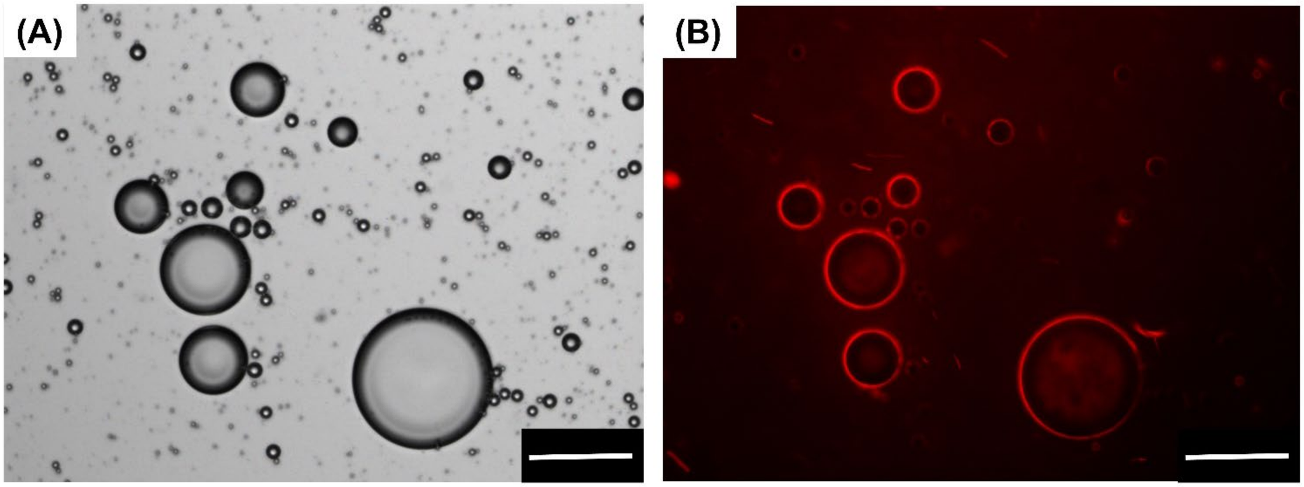

Supplementary Figure 2. Representative (a) bright field and (b) fluorescence image of hDox microbubbles showing co-localized hDox on the shell. The analysis was carried out on bubbles before isolation of nanobubbles to improve visualization of the bubble shell. Scale bar is 10 µm.

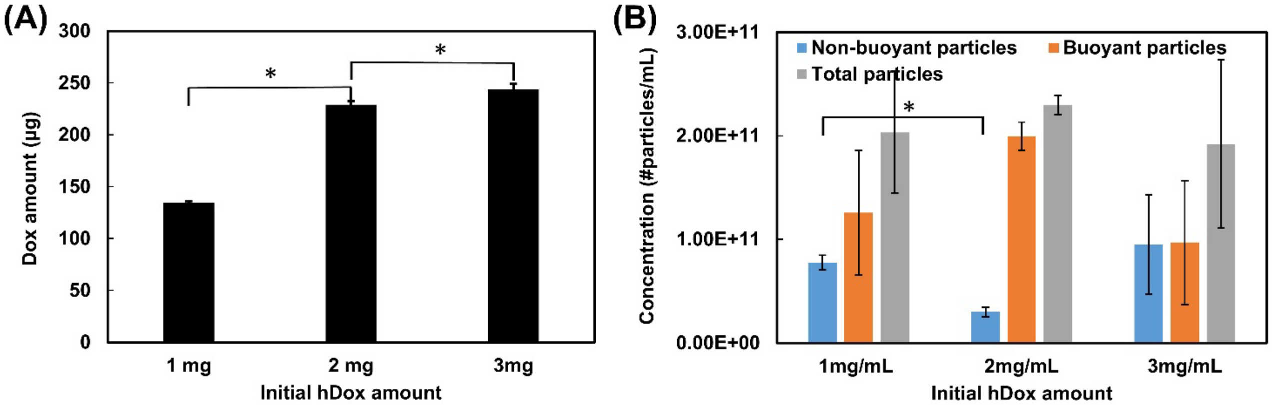

Supplementary Figure 3. Optimization of hDox-NB formulation (n=3). (a) Total mass of hDox extracted from NBs following loading at various initial hDox feeding concentration used in the bubble preparation; (b) Concentration of buoyant and non-buoyant particles measured by RMM for each hDox loading amount. 2 mg/mL loading was selected from this optimization because it provided both a high hDox content in NBs and a consistently high yield of buoyant particles in the total particle population (as shown in (b)). Error bars represent standard deviation. Asterisk indicates $p<0.05$.

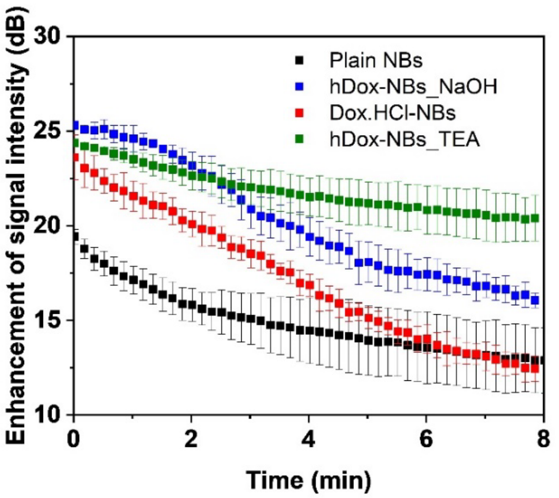

Supplementary Figure 4. Ultrasound signal decay of hDox-NBs (n=3) made from Dox that was deprotonated using either triethylamine or TEA (green squares) and NaOH (blue squares). The rate of decay of ultrasound signal from NBs made with TEA-deprotonated Dox was significantly slower than that of either plain NBs, Dox.HCl-NBs or NBs loaded with hDox deprotonated with NaOH. Error bars represent standard deviation.

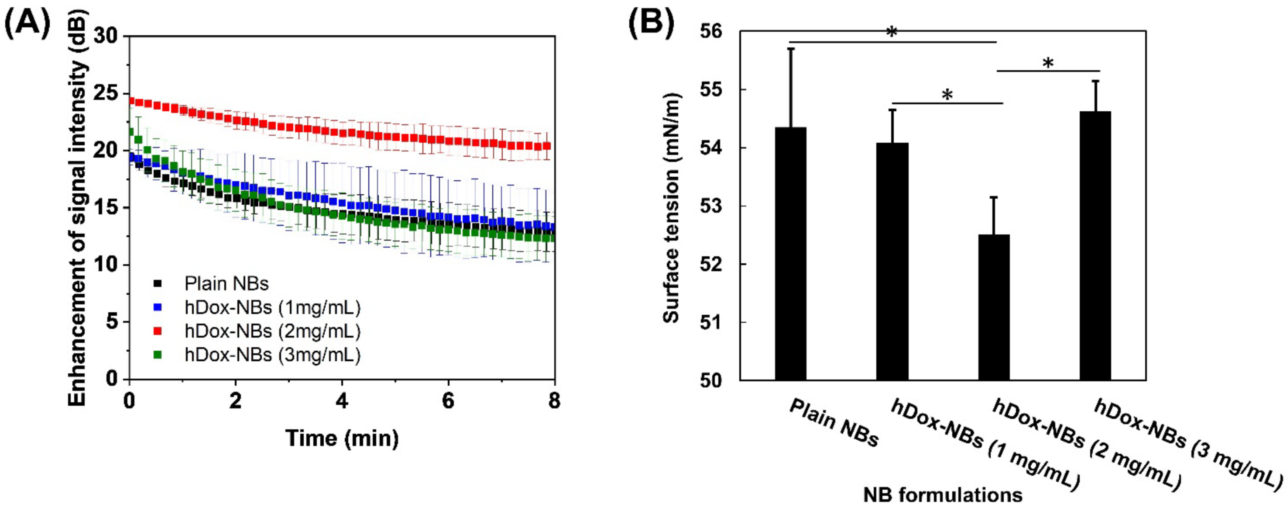

Supplementary Figure 5. (a) Ultrasound signal decay of hDox-NBs (n=3) at various initial hDox concentration; (b) Surface tension measurements of NB formulations with increasing amounts of hDox. The measurements were acquired using a pendant drop tensiometer method and were repeated in triplicate. Error bars represent standard deviation. Asterisk indicates $p<0.05$. The optimized NB formulation has slowest signal decay rate and the lowest surface tension.

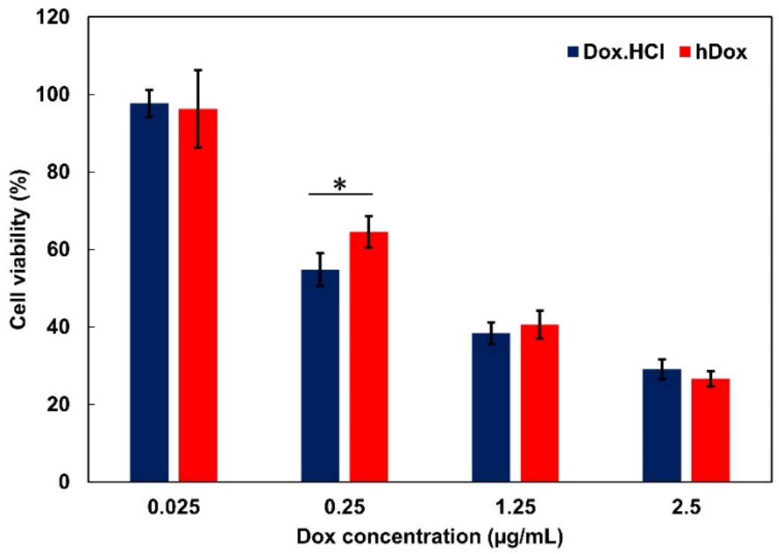

Supplementary Figure 6. Cytotoxicity of doxorubicin hydrochloride (Dox.HCl) and hydrophobic deprotonated Dox (hDox) in OVCAR-3 cells evaluated 72h after treatment. Assays were carried out in triplicate. Error bars represent standard deviation. No difference was seen between the hDox and Dox.HCl treatment groups.

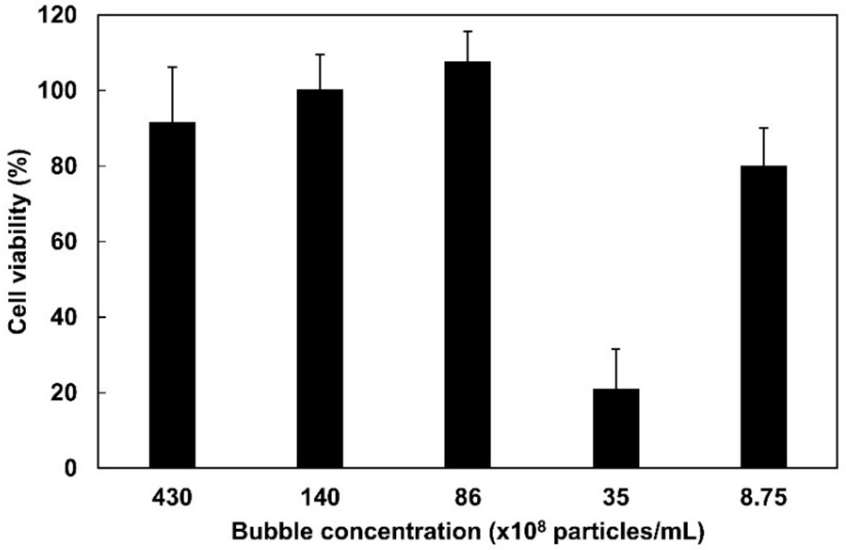

Supplementary Figure S7. Viability of OVCAR-3 cells following exposure to plain NBs and ultrasound at various NB concentrations. The WST-1 viability testing was perfromed 72h after treatment. All of the experiments were carried out in triplicate. Error bars represent standard deviation. Control plain NBs at 8.75 x10^8 particles/mLwhich is equivalent to the concentration of hDox-NBs used in cell viability experiments has cell viability at 80%.
